# SERPINE1 associated with remodeling of the tumor microenvironment in colon cancer progression: a novel therapeutic target

**DOI:** 10.1186/s12885-021-08536-7

**Published:** 2021-07-03

**Authors:** Shaokun Wang, Li Pang, Zuolong Liu, Xiangwei Meng

**Affiliations:** 1 Department of Emergency, The First Hospital of Jilin University, Changchun, China; 2 Department of Gastrointestinal Medicine, The First Hospital of Jilin University, No. 71 Xinmin Road, Changchun, China

**Keywords:** Colon cancer, SERPINE1, Tumor microenvironment, Tumor-infiltrating immune cells

## Abstract

**Background:**

The change of immune cell infiltration essentially influences the process of colorectal cancer development. The infiltration of immune cells can be regulated by a variety of genes. Thus, modeling the immune microenvironment of colorectal cancer by analyzing the genes involved can be more conducive to the in-depth understanding of carcinogenesis and the progression thereof.

**Methods:**

In this study, the number of stromal and immune cells in malignant tumor tissues were first estimated by using expression data (ESTIMATE) and cell-type identification with relative subsets of known RNA transcripts (CIBERSORT) to calculate the proportion of infiltrating immune cell and stromal components of colon cancer samples from the Cancer Genome Atlas database. Then the relationship between the TMN Classification and prognosis of malignant tumors was evaluated.

**Results:**

By investigating differentially expressed genes using COX regression and protein-protein interaction network (PPI), the candidate hub gene serine protease inhibitor family E member 1 (SERPINE1) was found to be associated with immune cell infiltration. Gene Set Enrichment Analysis (GSEA) further projected the potential pathways with elevated SERPINE1 expression to carcinogenesis and immunity. CIBERSORT was subsequently utilized to investigate the relationship between the expression differences of SERPINE1 and immune cell infiltration and to identify eight immune cells associated with SERPINE1 expression.

**Conclusion:**

We found that SERPINE1 plays a role in the remodeling of the colon cancer microenvironment and the infiltration of immune cells.

**Supplementary Information:**

The online version contains supplementary material available at 10.1186/s12885-021-08536-7.

## Background

Malignant tumors are characterized as exhibiting unlimited multiplication, evasion from growth and evading immune destruction [1], all of which are pathogenically related to the tumor microenvironment (TME) [2]. A healthy microenvironment inhibits carcinogenesis and metastasis, whereas a cancerous microenvironment may promote neoplastic development [3]. The TME consists of a complex network of various intracellular and extracellular components that play an indispensable role in cancer development and progression.

As a component of TME, immune and inflammatory cells have been shown to be closely associated with carcinogenesis. Inflammation has also been reported to be an important risk factor contributing to cancer development [4–6]. It is thought that chronic inflammation, tumor-related inflammatory responses, and inflammation in the tumor environment in the context of intestinal dysfunction contribute to the carcinogenesis of intestinal malignancies [7–9]. Immuno-inflammatory cell dynamics persist in the site of chronic inflammation, which has been proposed as the cradle for cancer development and progression [6, 10, 11]. Therefore, the association between inflammation and immune cells can reflect the relationship of carcinogenesis and prognosis of patients [12].

The role of immune cell infiltration and the differentially expressed genes associated with the infiltration in the remodeling of the colorectal cancer microenvironment has been of growing interest in the medical and scientific communities. To gain a more fundamental understanding of the molecular mechanism of TME remodeling in colon cancer progression, we propose here a computerized bioinformatics tool for identifying a candidate gene(s) from the Cancer Genome Atlas (TCGA) with regulatory functions in tumorigenesis.

## Methods

### Working samples

The transcriptome from the RNA-seq analysis of 524 colon cancer samples, including 42 normal samples and 482 tumor samples, with corresponding clinicopathological information were download from the TCGA database (https://portal.gdc.cancer.gov/). We employed the ESTIMATE algorithm to calculate the ImmuneScore, Stromalscores, and ESTIMATEScore for each sample in the tumor microenvironment.

### Survival analysis

After sorting the clinical data downloaded from the TCGA database, complete survival information of 455 cases was obtained with survival time ranging from 0 to 12 years. A Kaplan–Meier test was applied to plot the survival curve, while A log-rank test was used to compare the median of the survival times for the two different groups. A *p* value < 0.05 was considered statistically significant.

### Differential expression analysis

All patients were divided into a high and low score group based on the median values of the ImmuneScore and StromalScore. The linear models for the microarray data (LIMMA) package were further utilized for the differential analysis of gene expression. In comparing the two groups, the differentiation of gene expression of each group with more than a one-fold change following a log_2_ transformation was considered statistically significant at a *p* value threshold of 0.05 after false discovery rate (FDR) correction. The differentially expressed genes were plotted as heat maps using the Heat map package of R software.

### GO and KEGG enrichment analysis

The genes obtained through the differential expression analysis were further analyzed with the R software using the clusterProfiler, enrichplot, and ggplot2 packages to identify those that were significantly enriched [13]. Significance thresholds were set a 0.05 for both p and q value.

### Differential analysis of scores with clinical stages

Clinic-pathological data of the colon cancer samples were obtained from TCGA and further analyzed with the R software package. A Wilcoxon rank-sum or Kruskal–Wallis rank-sum test was used for establishing significance.

### Construction of PPI network

The STRING database was used to predict a PPI, which was reconstructed with the Cytoscape v3.6.1 software. The connectional nodes for constructing the network were the ones with a confidence of interactive relationship of more than 0.95.

### COX regression analysis

Univariate COX regression was performed with the R software. With *p* values from the Cox regression analysis, the top 24 genes were plotted according to a ranking from small to large.

### Gene set enrichment analysis

The KEGG pathway gene set (C2.cp.kegg.v7.1.symbols.gmt) was acquired from the Molecular Signatures Database (MSigDB) as the target set. Whole transcriptomes of all tumor samples underwent gene set enrichment analysis (GSEA) using the gsea-3.0 software from Broad Institute. Through GSEA, the gene sets with NOM *p* < 0.05 and FDR q > 0.06 were processed for the next round of analyese.

### Immune cell infiltration

The CIBERSORT computational algorithm was applied for estimating the abundance of immune cell infiltration in all tumor samples. Candidate tumor samples with *p* < 0.05 were identified for more detailed analysis.

### Statistical analysis

All statistical analyses were performed with the R software (version 3.5.2). A Student’s t-test was used to compare the differences between the two variables and a two-tailed *p* < 0.05 was considered statistically significant.

## Results

### Relationship between immune cell infiltration and tumor prognosis and staging

**Fig. 1:**
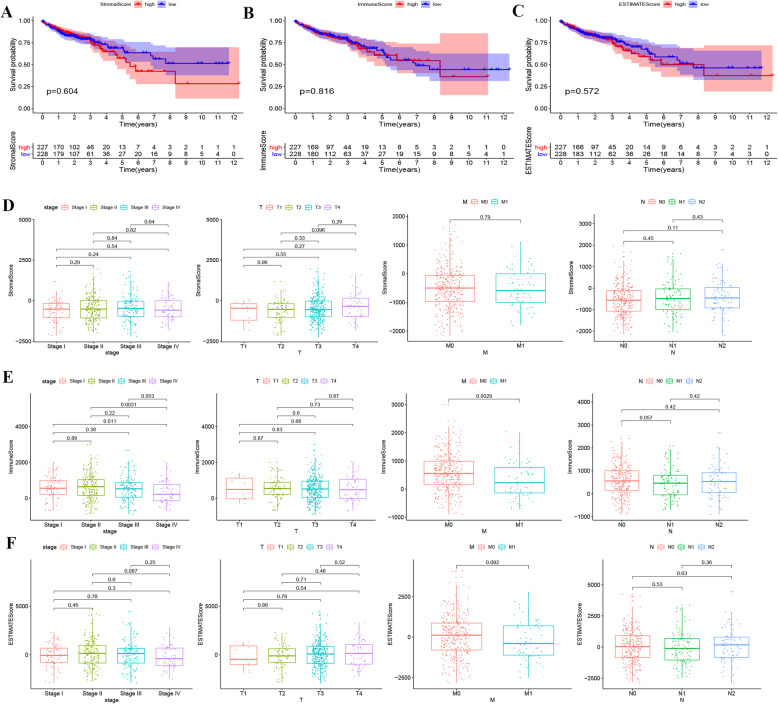
Relationship between immune score and colon cancer prognosis and staging. **A**, **B**, **C** Kaplan-Meier Survival curves for colon cancer patients with high and low StromalScores (ImmuneScore, ESTIMATEScore). The median of the survival times for both groups was compared using a log-rank test with *p* values of 0.604 (0.816, 0.572, respectively). **D** Distribution of StromalScore in stage and TMN. The p values were all greater than 0.05, according to a Kruskal-Wallis rank sum test. **E** The distributed patterns of immune cells in colorectal cancer were significantly different between stage I vs IV and stage II vs IV with p values of 0.011 and 0.0031, respectively. The distribution of immune cells was different between pM0 vs pM1 (*p* = 0.0029), but no difference between the pT_1–4_ and pN_0–2_ stages was observed when using a Kruskal-Wallis rank-sum test. **F** Distribution of ESTIMATEScore in stage and TMN. The p values were all greater then 0.05, according to a Kruskal-Wallis rank sum test

We first scored the immune and stromal cells of colon cancer samples with the scoring system shown in Table S1. The sample scores were then paired with the corresponding clinical information. Next, with a Kaplan-Meier test, we determined the survival curves for colon cancer patients with a high or low StromalScore, ImmuneScore, or ESTIMATEScore, which were then statistically analyzed for survival rates with a *P* value of 0.604, 0.816, and 0.572, respectively (Fig. 1A-C). Moreover, the StromalScore and ESTIMATEScore did not show a difference between Stage and TMN (Fig. 1D, F), but the ImmuneScore did show a significant difference (*P* < 0.05) between stages I and IV, stages II and IV, and between M0 and M1 (Fig. 1E). Additionally, we calculated the survival difference between the various groups with high and low ImmuneScores for intestinal adenocarcinoma and found that *P* values were greater than 0.05 (Figure S1).

### Enrichment of genes associated with immune cell infiltration

**Fig. 2:**
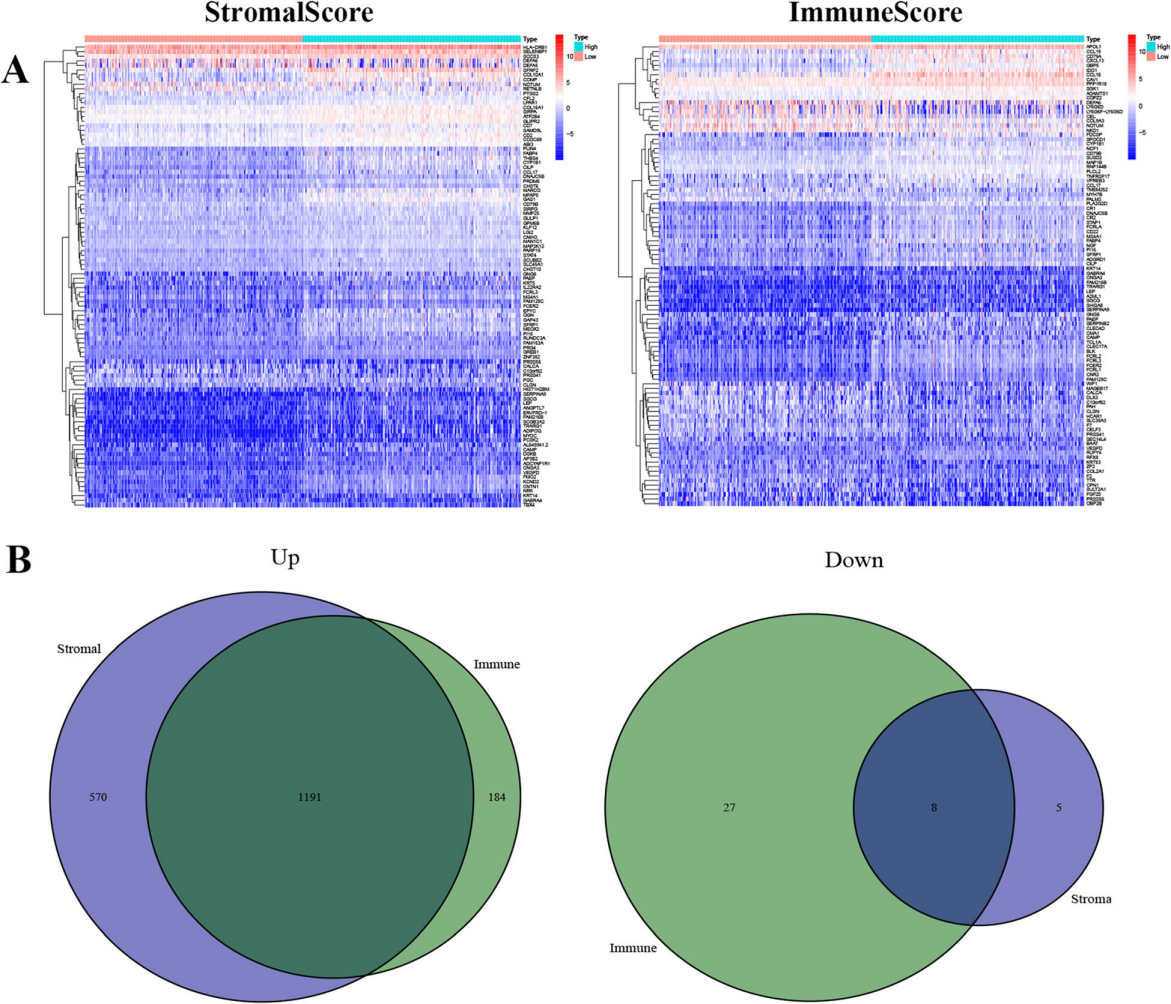
The differential expression of genes in infiltrating stromal and immune cells. **A** Heatmap displaying the top 50 genes among those exhibiting differences between high and low StromalScore or ImmuneScore groups. **B** Ven diagram displaying the intersection of up and downregulated genes with differential expressions influenced by StromalScore apart from ImmuneScore (q < 0.05 and fold change more than one after log_2_ transformation)

**Fig. 3:**
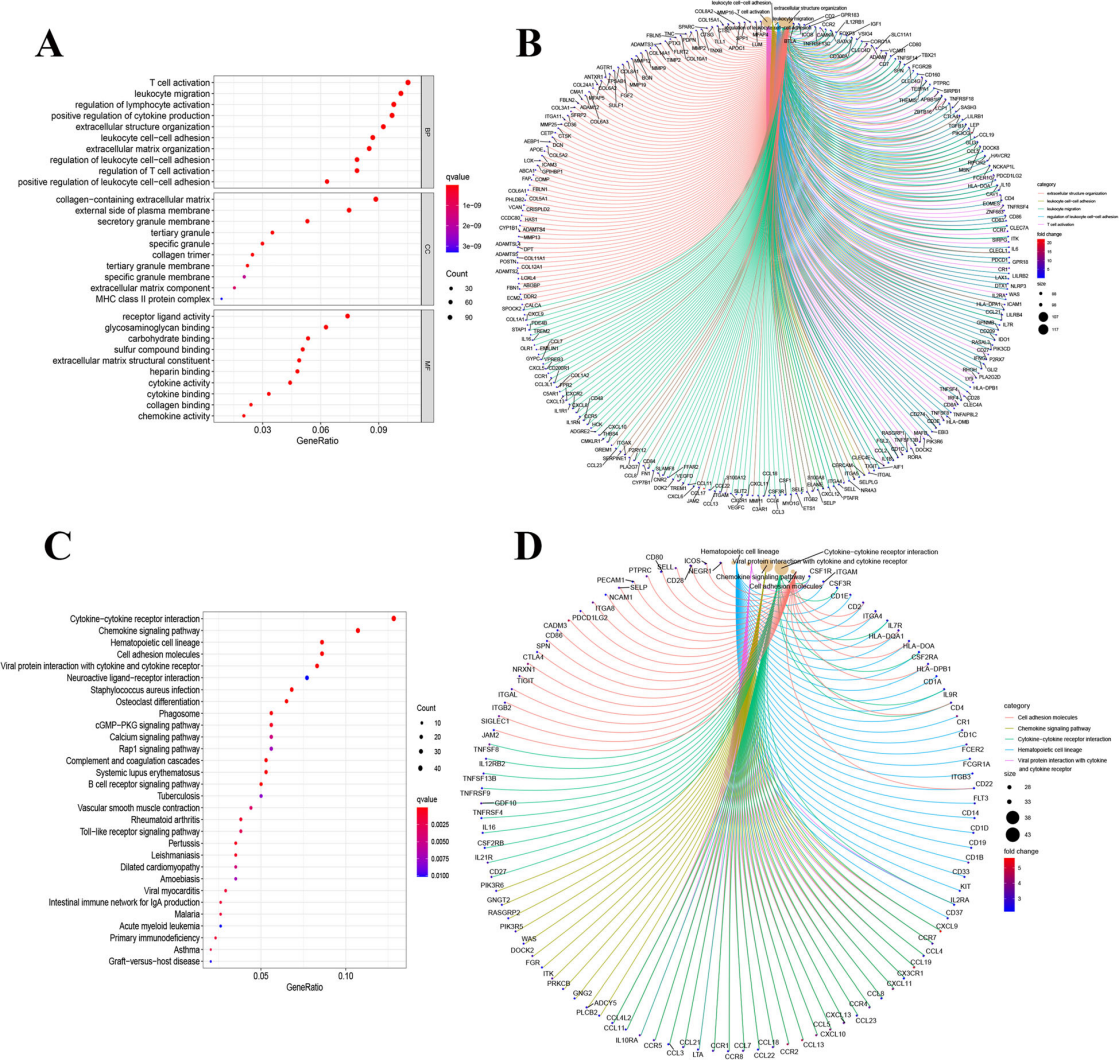
Enrichment genes. **A**, **B** GO enrichment analysis for 1199 differentially expressed genes (both p and q < 0.05 indicates significant enrichment); **C**, **D** KEGG enrichment analysis for 1199 differentially expressed genes, (p and q < 0.05 indicates significant enrichment)

Among the genes associated with stromal cell invasion, 1761 up-regulated and 13 down-regulated genes were identified, while 1375 up-regulated and 35 down-regulated genes were related to immune cell invasion. The top 50 genes most likely related to stromal cell and immune cell infiltration are displayed in Fig. 2A. When examining the intersecting of genes related to stromal and immune cell infiltration, 1191 up-regulated genes and 8 down-regulated genes were related to both (Fig. 2B). Gene ontology (GO) enrichment analysis was first performed on these 1199 genes, which were mainly enriched and correlated to the pathways of T cell activation, leukocyte migration, regulation of lymphocyte activation and other functions (Fig. 3A, B). Secondly, the Kyoto Encyclopedia of Genes and Genomes (KEGG) enrichment analysis was also performed on both up- and down-regulated genes with a priori functions of cytokine-cytokine receptor interaction, chemokine signaling pathway, and others (Fig. 3C) for establishing the correlation (Fig. 3D). Therefore, by using two gene-enrichment analysis methods, the genes exhibiting immune-related factors in the immune microenvironment of colorectal cancer were identified.

### Screening of immune-related genes in colon cancer

**Fig. 4:**
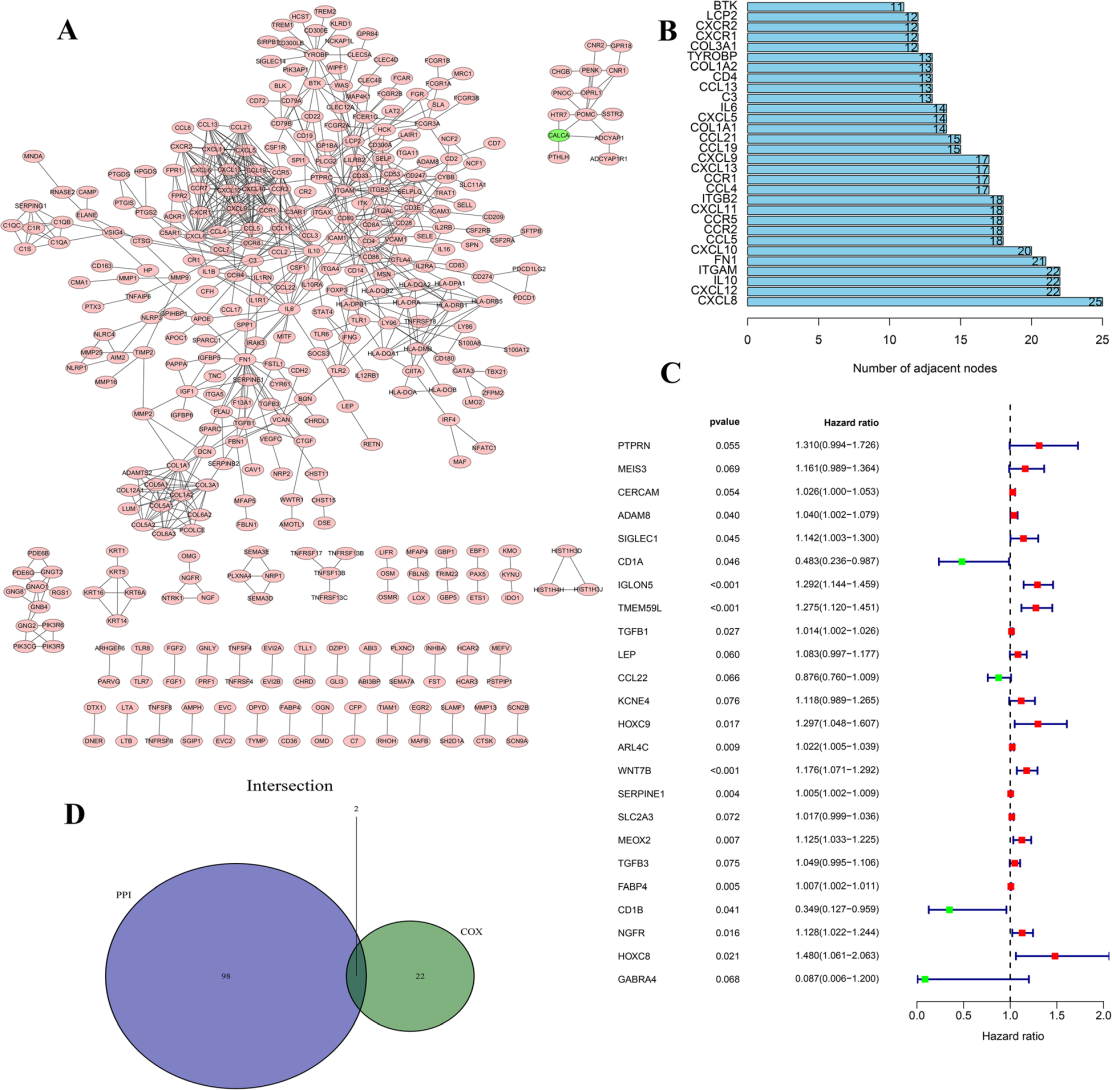
Screening of immune-related proteins. **A** A protein-protein interaction network (PPI) was constructed with an interaction confidence value > 0.95. **B** The top 30 genes with the most nodes and their sub-nodes. **C** Following univariate COX regression analysis on 1199 genes, a list of the candidate genes is displayed using a significance factor of *p* < 0.1. **D** Venn diagram showing the hub genes after the top 100 genes from PPI were matched to the top 24 genes as determined by the univariate COX regression

Through our computational analysis, 1199 genes were identified as being associated with immune cell and stromal cell infiltration. We first used String to demonstrate the interaction of gene-related proteins (Figure S2). We then used the Cytoscape software to visualize the interactional network of the proteins (Fig. 4A), which accentuated the number of nodes. Afterward, the hub genes were selected as the ones with the most nodes in the networks, among which we displayed first 30 in Fig. 4B. Furthermore, we also applied univariate COX regression to analyze the potential contribution of the differential expression of all the genes to the survival of CRC patients, and eventually obtained 24 candidate genes (Fig. 4C). The 100 genes with the most nodes in the PPI were combined with the top 24 genes ranked by *p*-value in the univariate COX regression analysis to finally identify the two genes TGFB1 and SERPINE1 (Fig. 4D).

### The association of SERPINE1 expression with TMN classification and colon cancer prognosis

**Fig. 5:**
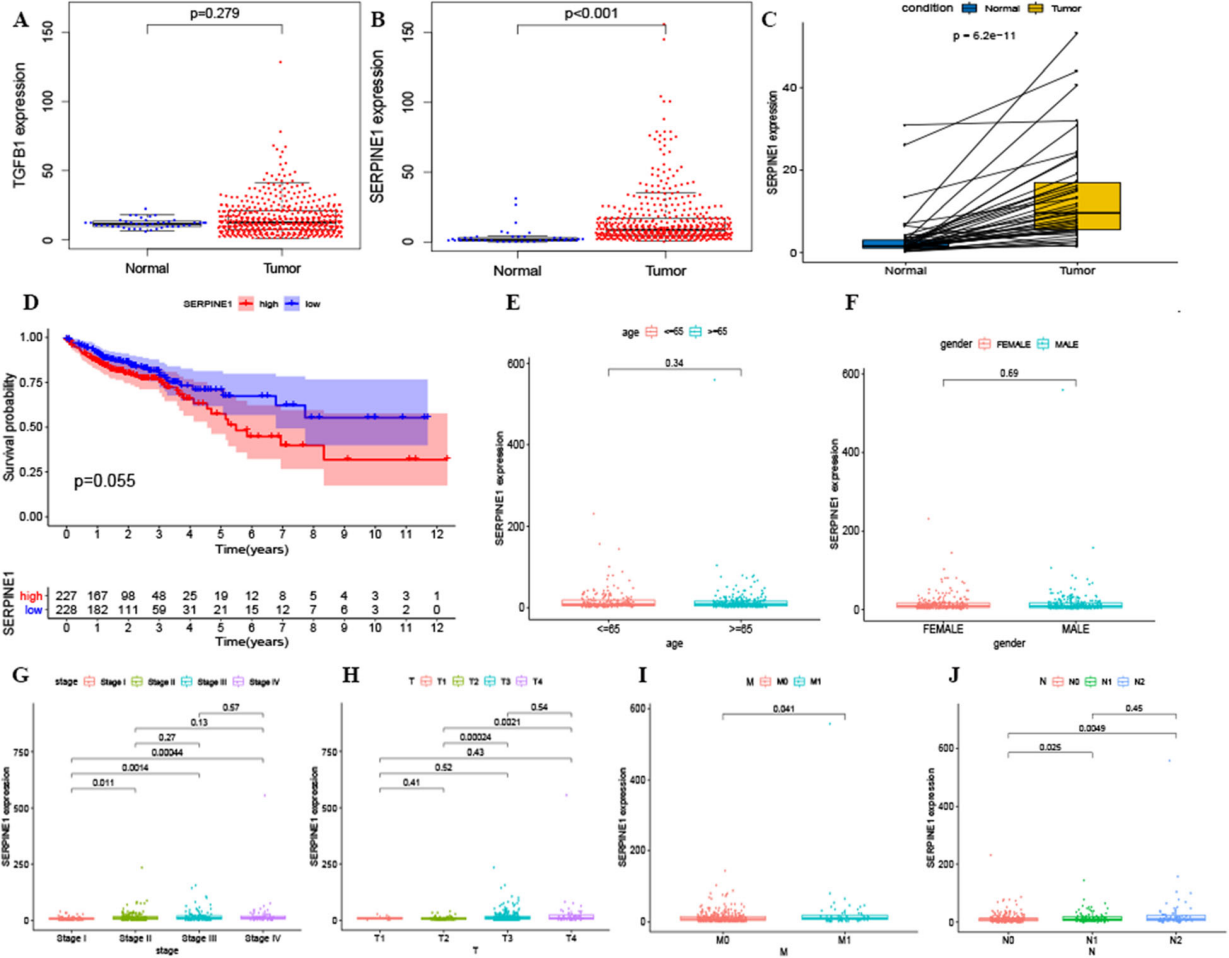
Genes with differential expressions in colorectal cancer patients, and the relationship between SERPINE1 expression and cancer prognosis and staging. B TGFB1 was not significantly expressed in normal and tumor samples, *p* > 0.05. **B** SERPINE1 shows differential expression in normal and colon cancer samples, *p* < 0.05. **C** Paired analysis of SERPINE1 expression in normal and colon cancer samples, p < 0.05. **D** Effect of SERPINE1 on the prognosis of colorectal cancer patients. The patients were further divided into two groups based on high or low expression of SERPINE1 according to the median value (assessed using a log-rank test with *p* = 0.055). **E-J** The correlation of the expression of SERPINE1 with clinicopathological staging. A Wilcoxon rank-sum or Kruskal-Wallis rank-sum test was utilized

From the previous experiment, the TGFB1 and SERPINE1 were further analyzed concerning their expression in colorectal cancer and normal tissue. The results showed that while SERPINE1 expression was significantly different between colorectal cancer and normal tissue, TGFB1 expression was not (Fig. 5A, B). Interestingly, SERPINE1 manifested with differential expression in paired cancerous and adjacent tissues (Fig. 5C, *p* < 0.05). The patients with colon cancer were then divided into two groups based on either high or low expression according to the median value of SERPINE1 expression, however no difference in prognosis was observed between the two groups (*p* = 0.055) (Fig. 5D).

Moreover, SERPINE1 was compared with other tumor-related factors and was found to be dissociated with age and gender (Fig. 5E and F, *p* > 0.05). Furthermore, the expression of SERPINE1 in stage I CRC was significantly different from that of the other three stages (Fig. 5G, *p* < 0.05). Following this stratification, SERPINE1 expression at pT2 was significantly different from that of pT3 and pT4 (Fig. 5H, *p* < 0.05). At pN0, is significant difference was found between pN1 and pN2 (Fig. 5J, *p* < 0.05), however, pM0 was significantly different from pM1 (Fig. 5I, *p* < 0.05).

### The regulatory role of SERPINE1 in the immune microenvironment

**Fig. 6:**
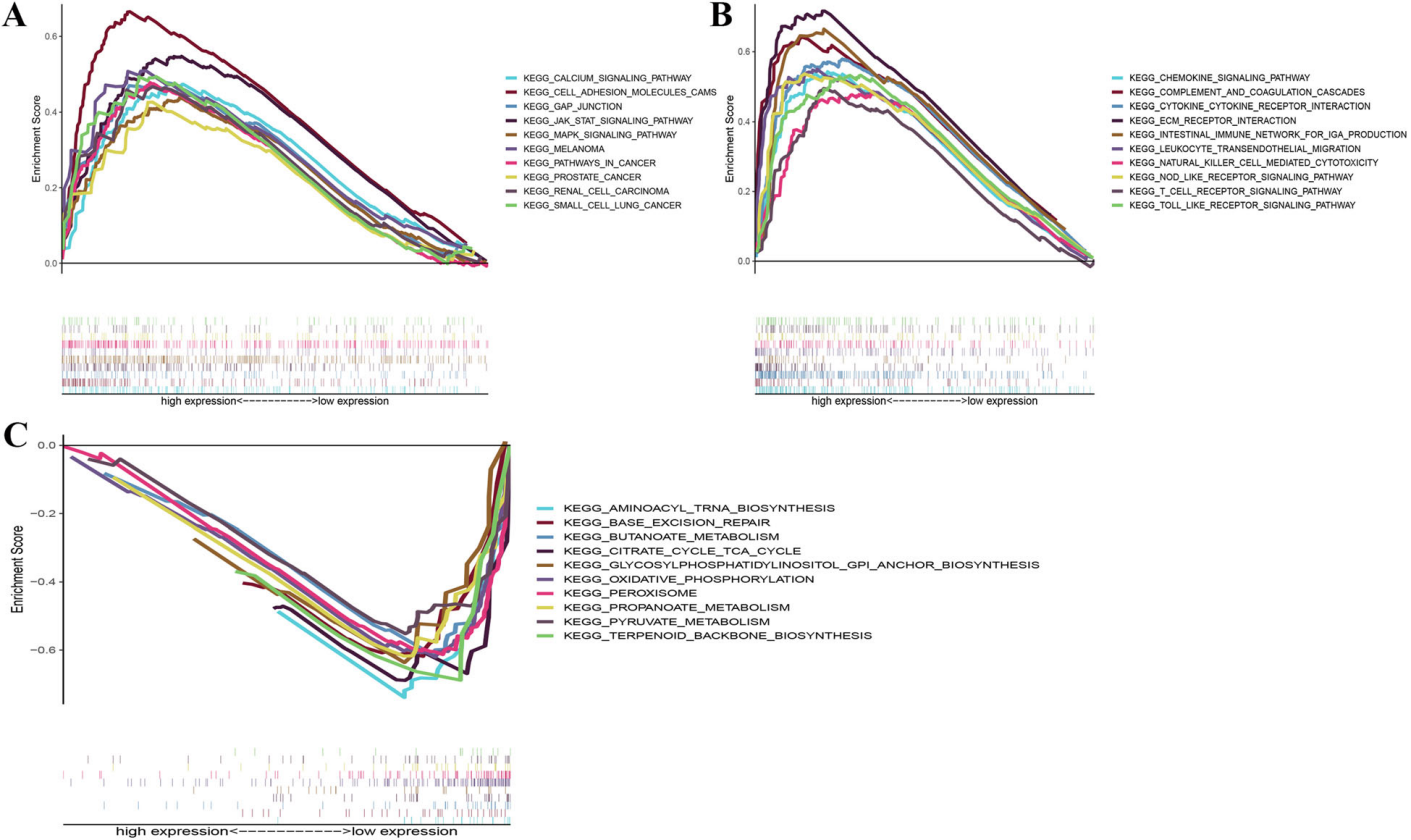
Enrichment of SERPINE1 and the related pathways by GSEA. **A** Enrichment of highly expressed SERPINE1 in tumor-associated pathways. **B** Enrichment of highly expressed SERPINE1 in immune-related pathways. **C** Low expression of SERPINE1 enrichment. (Only gene sets with NOM *p* < 0.05 as well as FDR q < 0.05 were considered statistically significant)

GSEA was used to analyze the effects of enrichment between SERPINE1 and various cancer related pathways (Fig. 6A). The enrichment of groups with high expression of SERPINE1 predominantly converged into carcinogenesis-related pathways and various immune-related pathways that included the chemokine signaling pathway, the cytokine receptor interaction, the intestinal immune network for IgA production and T cell receptors (Fig. 6B, Table S2). For the group with low expression of SERPINE1, genes were enriched in metabolism and oxidative phosphorylation pathways (Fig. 6C, Table S3).

### Relationship between SERPINE1 and immune cell infiltration

**Fig. 7:**
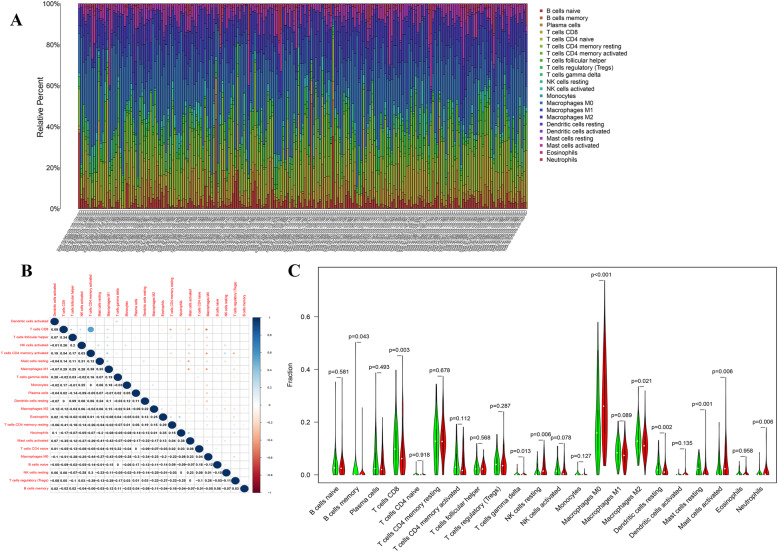
Immune cell profile in colon cancer samples and correlation analysis. A bar plot displaying the proportion of 22 different immune cells in colon cancer samples (column names are sample IDs). **B** Bubble chart displaying the correlation between each of 22 different immune cells and the number in each bubble, calculating the *p*-value of the correlation between two kinds of cells. The shade of each color bubble represents the corresponding correlation value between two cells, and the Pearson coefficient was used as the significance test. **C** Violin plot showing the differentiation ratio of 22 different immune cells between colon tumor samples with low or high median SERPINE1 expression. A Wilcoxon rank-sum was used as the significance test

**Fig. 8:**
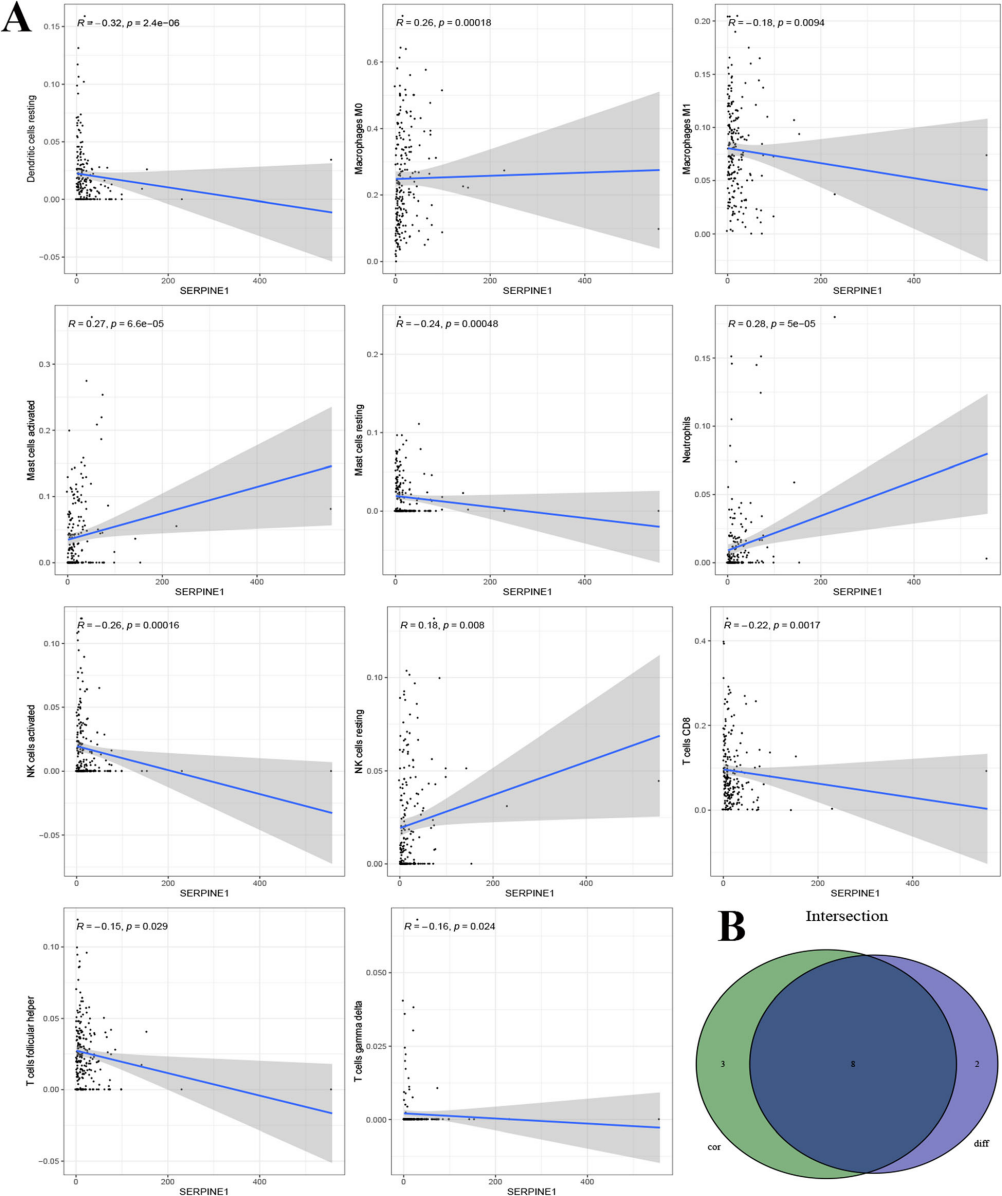
Correlation of the proportion of immune cells with SERPINE1 expression. A Scatter plot showing the correlation between the proportion of 11 different immune cells and SERPINE1 expression (*p* < 0.05). The blue line in each plot is consistent with a linear model, indicating that the correlation test is based on the proportion tropism of the immune cell along with SERPINE1 expression. **B** Venn diagram displaying eight different immune cells that correlate with SERPINE1 expression codetermined by difference and correlation tests displayed in previous violin and scatter plots, respectively

To further understand the effect of SERPINE1 on immune cell infiltration, the CIBERSORT algorithm was used to construct 22 immune cell profiles in colon cancer samples (Fig. 7A). First, the relationship between 22 selected immune cells were shown in Fig. 7B, and were further analyzed by dividing the samples into two groups with high and low expression of SERPINE1. This was performed to compare the infiltration of immune cells between the high and low expression groups, resulting in the identification of 12 immune cells with obvious differences (Fig. 7C). Secondly, the corresponding graphs of gene expression and immune cell content for each sample were plotted to unveil the correlation between the SERPINE1 expression and immune cell content (Fig. 8A). This analysis subsequently identified eight immune cells, including T cells CD8, T cells gamma delta, NK cells resting, Macrophages M0, Dendritic cells resting, resting Mast cells, activated Mast cells and Neutrophils (Fig. 8B).

## Discussion

The relationship between immune cell infiltration and cancer development has been widely reported in literature [14–16]. However, the correlation between immune cell infiltration and tumor prognosis remains controversial for colorectal cancer. Studies have shown that the poor prognosis of colon cancer is either positively or negatively interdependent with tumor-associated neutrophils [17, 18], and Some studies have shown that Tregs can promote the prognosis of CRC [18, 19], while others have identified Tregs as a risk factor for CRC [20, 21]. Other immune cells have also been reported [22, 23]. Moreover, some studied reported that adipocytes in stromal cells can induce epithelial mesenchymal differentiation of tumors, subsequently promoting tumor metastasis [24]. By contrast, some factors secreted by stromal cell might also regulate tumor cell metastasis, apoptosis and other processes [25, 26]. However, this study did not prove the association of stromal cells with prognosis, stage and TMN of the CRC patients, and also rejected the impact of immune cells on tumor microenvironment in our study. Ye L et al. also performed a similar analysis with 1008 colon cancer samples from both TCGA and GEO databases and suggested the association of immune cell infiltration with the prognosis of colon cancer [22]. For this inconsistence, possible explanations could be due to the differences in database selection and sample size. Besides, immune and stromal cells contain numerous cell types, so the influence of the immune microenvironment on prognosis may vary from the perspective of these different cell types. More importantly, colorectal cancer cell types possess diversity, which might respond differently to any given immune microenvironment.

In addition to survival analysis, TMN classification is commonly applied in the clinic to assess tumor progression. Some studies have suggested that this classification does not account for immune status, so response to treatment may not be an effective predictor [27]. Of course, some studies have also found that tumor-associated neutrophils, regulator T cells and tumor-associated macrophages are associated with undifferentiated colorectal cancer with advanced TMN classification [28, 29]. Our study found that differences in the degree of infiltration of immune cells is a strong biomarker for classifying stage II vs stage III/IV, pN0 vs pN1, and pM0 vs pM1. We also found that even though the immune microenvironment is influenced by both stromal and immune cells, no difference existed in the pT, pM, and pN classifications, except for differences between stage II vs stage IV. This suggests that components in the tumor microenvironment possess different and refined functions.

Changes in the tumor microenvironment are determined by genes [30]. For identifying the gene(s) associated with the remodeling of the cancerous microenvironment in the colon cancer progression, we propose here an algorithm for pipelining numerous available bioinformatics tools. This pipeline aims to analyze the genes with differential expressions that are congruous with the differences in the infiltration of stromal and immune cells. In short, GO enrichment analysis coupled with KEGG enrichment analysis identifies the genes relevant for immune-related factors in the immune microenvironment of colorectal cancer. Then, STRING coupled with Cystoscape constructs the involved genes into a PPI, which is coupled with Cox univariate regression analysis to predict the most likely candidate genes.

We first analyzed the GO data and found that most of the differentially expressed genes were related to the activation of T cells, migration of leukocytes and regulation of lymphocytes. Through KEGG enrichment analysis, the candidate genes were mainly related to the cytokine receptor interaction and chemokine signaling pathway, which are also immune-related pathways. Taken together, the regulatory role of genes in the immune microenvironment was confirmed. This finding is also consistent with the views of David Tamborero and other scholars [30]. Eventually. The PPI and Univariate COX regression analysis were used to identify the two genes TGFB1 and SERPINE1. The comparison between cancer and normal samples showed no significant difference in TGFB1 expression; however, SERPINE1 expression was significantly different between cancer and normal samples, but had no significant effect on prognosis [31]. The inhibitory effect of SERPINE1 expression on tumor cell apoptosis has been previously reported [32, 33]. However, the relationship between SERPINE1 and immunity has been studied much less. We used GSEA to analyze the relationship between SERPINE1 expression and cancerous pathways and found that high SERPINE1 expression can promote tumor and immune-related pathway activation. This suggests that SERPINE1 can influence the occurrence and development of colon cancer by regulating the tumor immune microenvironment.

SERPINE1, known as the Serine Protease Inhibitor family E member 1 or plasminogen activator inhibitor-1 (PAI-1), has been proposed as the key player for carcinogenesis and poor prognosis [32–34]. In previous studies, SERPINE1 promoted peripheral neo-angiogenesis, regulated endothelial homeostasis, and interacted with inflammatory factors [33, 35, 36], suggesting that SERPINE1 may be related to the tumor microenvironment. However, the role of SERPINE1 in the tumor microenvironment with immune-related cells has not been reported in previous studies.

The role of SERPINE1 in the process of skin fibrosis has also been reported [37]. Studies have shown that SERPINE1 plays multiple critical roles as a mediator of infiltration, adhesion, and activation of mast cells and fibroblasts in fibrogenesis. In the process of renal fibrosis, the decrease of SERPINE1 expression is also associated with the decrease of neutrophils and macrophages [38, 39], suggesting that SERPINE1 can act as a chemokine that interacts with other immune cells. Therefore, to confirm whether SERPINE1 can act on other immune cells in the tumor immune microenvironment, CIBERSORT was used to assess the relationship between the expression differences of SERPINE1 and immune cell infiltration. Here, we identified 10 immune cells with the most obvious differences and further analyzed the correlation of the proportion of 11 kinds of tumor-infiltrating immune cell with SERPINE1 expression. Through the intersection of these two groups, we finally identified eight immune cell types that included neutrophils, mast cells, and macrophages, which have been reported in other diseases. Meanwhile, this group also contained T cells CD8, T cells gamma delta, NK cells and dendritic cells.

## Conclusion

In conclusion, through our proposed algorithm, various computer-based bioinformatics platforms and tools were used to extract a list of immune microenvironment-related genes of prognostic value for colon cancer. We further identified SERPINE1as a potential immune cell infiltration regulator that can interact with eight immune cell types for the remodeling of the tumor microenvironment for colon cancer development and progressions.

## Supplementary Information

**Additional file 1.**

**Additional file 2: Figure S1.** The scores of stromal and immune cells in intestinal adenocarcinoma were compared. No correlation was found. **Figure S2.** PPIN built from the STRING database. **Table S2** Enrichment of related pathways in the group with highly expressed SERPINE1 gene. **Table S3** Enrichment of related pathways in the group with lowly expressed SERPINE1 gene.

## Data Availability

The datasets generated and/or analysed during the current study are available in the TCGA repository, https://portal.gdc.cancer.gov/

## Abbreviations

PPI: Protein-protein interaction network
SERPINE1: Serine protease inhibitor family E member 1
GSEA: Gene Set Enrichment Analysis
TME: Tumor microenvironment
TCGA: The Cancer Genome Atlas
LIMMA: Linear models for the microarray data
FDR: False discovery rate
MSigDB: Molecular Signatures Database
GO: Gene ontology
KEGG: Kyoto Encyclopedia of Genes and Genomes.
