# Supplementary material for: SERPINE1 associated with remodeling of the tumor microenvironment in colon cancer progression: a novel therapeutic target
Source: BMC Cancer. 2021 Jul 3;21:767. doi: 10.1186/s12885-021-08536-7 (PMC8254339; doi:10.1186/s12885-021-08536-7)
Supplement: Supplementary file 2 — Additional file 2: Figure S1. The scores of stromal and immune cells in intestinal adenocarcinoma were compared. No correlation was found. Figure S2. PPIN built from the STRING database. Table S2 Enrichment of related pathways in the group with highly expressed SERPINE1 gene. Table S3 Enrichment of related pathways in the group with lowly expressed SERPINE1 gene. [file 12885_2021_8536_MOESM2_ESM.docx]

Supplementary Material


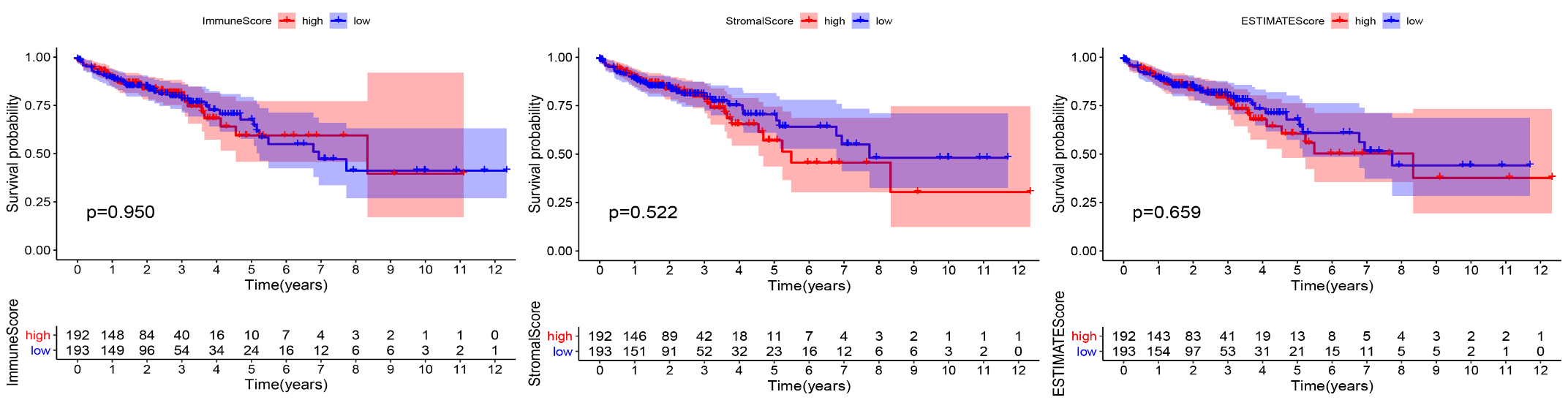


**Figure S1.** The scores of stromal and immune cells in intestinal adenocarcinoma were compared. No correlation was found.


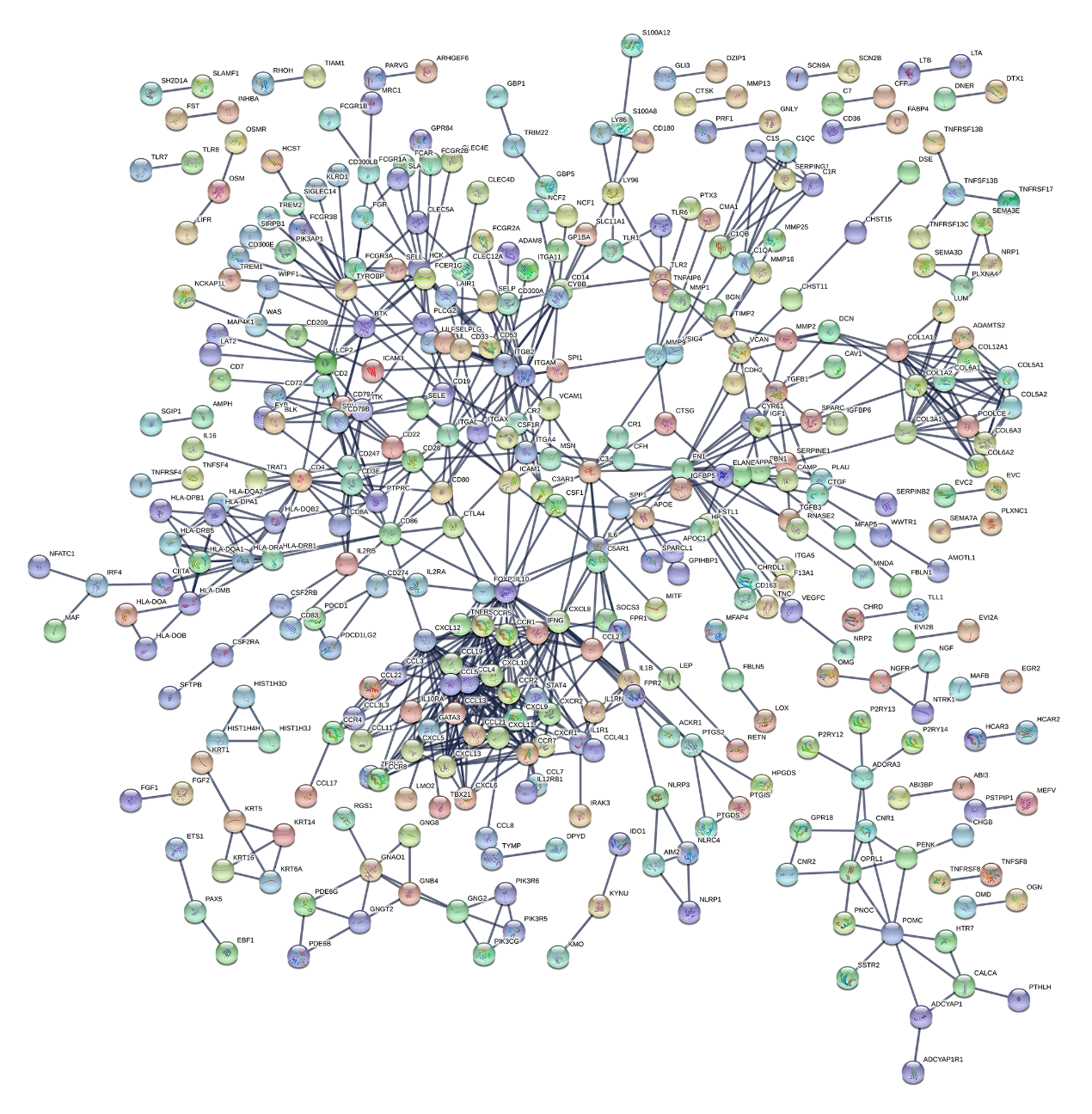


**Figure S2.** PPIN built from the STRING database

**Table S2** Enrichment of related pathways in the group with highly expressed SERPINE1 gene

| NAME | NOM p-val | FDR q-val | FWER p-val |
| --- | --- | --- | --- |
| KEGG_FOCAL_ADHESION | 0 | 0 | 0 |
| KEGG_CELL_ADHESION_MOLECULES_CAMS | 0 | 0 | 0 |
| KEGG_CYTOKINE_CYTOKINE_RECEPTOR_INTERACTION | 0 | 0.00274 | 0.006 |
| KEGG_ECM_RECEPTOR_INTERACTION | 0 | 0.002055 | 0.006 |
| KEGG_CHEMOKINE_SIGNALING_PATHWAY | 0 | 0.002738 | 0.01 |
| KEGG_LEISHMANIA_INFECTION | 0 | 0.002856 | 0.013 |
| KEGG_REGULATION_OF_ACTIN_CYTOSKELETON | 0 | 0.00257 | 0.014 |
| KEGG_PATHWAYS_IN_CANCER | 0 | 0.003036 | 0.019 |
| KEGG_VIRAL_MYOCARDITIS | 0.002105 | 0.002804 | 0.02 |
| KEGG_LEUKOCYTE_TRANSENDOTHELIAL_MIGRATION | 0 | 0.002752 | 0.021 |
| KEGG_HEMATOPOIETIC_CELL_LINEAGE | 0 | 0.002839 | 0.025 |
| KEGG_COMPLEMENT_AND_COAGULATION_CASCADES | 0 | 0.002779 | 0.026 |
| KEGG_AXON_GUIDANCE | 0 | 0.004157 | 0.037 |
| KEGG_JAK_STAT_SIGNALING_PATHWAY | 0 | 0.004466 | 0.041 |
| KEGG_DILATED_CARDIOMYOPATHY | 0 | 0.006091 | 0.051 |
| KEGG_SYSTEMIC_LUPUS_ERYTHEMATOSUS | 0.006316 | 0.008278 | 0.068 |
| KEGG_TOLL_LIKE_RECEPTOR_SIGNALING_PATHWAY | 0 | 0.008571 | 0.074 |
| KEGG_HYPERTROPHIC_CARDIOMYOPATHY_HCM | 0 | 0.010274 | 0.092 |
| KEGG_MAPK_SIGNALING_PATHWAY | 0 | 0.012008 | 0.111 |
| KEGG_NOD_LIKE_RECEPTOR_SIGNALING_PATHWAY | 0.001972 | 0.013206 | 0.128 |
| KEGG_SMALL_CELL_LUNG_CANCER | 0 | 0.012634 | 0.128 |
| KEGG_MELANOMA | 0 | 0.016546 | 0.164 |
| KEGG_CALCIUM_SIGNALING_PATHWAY | 0.001848 | 0.018675 | 0.192 |
| KEGG_T_CELL_RECEPTOR_SIGNALING_PATHWAY | 0.007952 | 0.019289 | 0.205 |
| KEGG_ARRHYTHMOGENIC_RIGHT_VENTRICULAR_CARDIOMYOPATHY_ARVC | 0.001923 | 0.019824 | 0.213 |
| KEGG_VASCULAR_SMOOTH_MUSCLE_CONTRACTION | 0.001876 | 0.019402 | 0.214 |
| KEGG_GLYCOSAMINOGLYCAN_BIOSYNTHESIS_CHONDROITIN_SULFATE | 0.006237 | 0.019829 | 0.221 |
| KEGG_GAP_JUNCTION | 0.003781 | 0.01977 | 0.229 |
| KEGG_INTESTINAL_IMMUNE_NETWORK_FOR_IGA_PRODUCTION | 0.010395 | 0.023382 | 0.273 |
| KEGG_GRAFT_VERSUS_HOST_DISEASE | 0.02459 | 0.024539 | 0.289 |
| KEGG_NATURAL_KILLER_CELL_MEDIATED_CYTOTOXICITY | 0.006048 | 0.027197 | 0.319 |
| KEGG_RENAL_CELL_CARCINOMA | 0.004057 | 0.02757 | 0.329 |
| KEGG_GLYCOSAMINOGLYCAN_BIOSYNTHESIS_HEPARAN_SULFATE | 0.003922 | 0.027005 | 0.335 |
| KEGG_ASTHMA | 0.014463 | 0.026447 | 0.336 |
| KEGG_TYPE_I_DIABETES_MELLITUS | 0.02537 | 0.030923 | 0.378 |
| KEGG_AUTOIMMUNE_THYROID_DISEASE | 0.023762 | 0.033624 | 0.409 |
| KEGG_PROSTATE_CANCER | 0.008114 | 0.033249 | 0.414 |
| KEGG_ANTIGEN_PROCESSING_AND_PRESENTATION | 0.024691 | 0.032796 | 0.417 |
| KEGG_GLIOMA | 0.002049 | 0.041522 | 0.495 |
| KEGG_HEDGEHOG_SIGNALING_PATHWAY | 0.009452 | 0.044847 | 0.533 |
| KEGG_PRION_DISEASES | 0.016293 | 0.044908 | 0.543 |
| KEGG_NEUROACTIVE_LIGAND_RECEPTOR_INTERACTION | 0 | 0.044553 | 0.547 |
| KEGG_PANCREATIC_CANCER | 0.002079 | 0.044745 | 0.556 |
| KEGG_ALLOGRAFT_REJECTION | 0.046025 | 0.045353 | 0.573 |
| KEGG_TIGHT_JUNCTION | 0.001862 | 0.049094 | 0.6 |

**Table S3** Enrichment of related pathways in the group with lowly expressed SERPINE1 gene

| NAME | NOM p-val | FDR q-val | FWER p-val |
| --- | --- | --- | --- |
| KEGG_PEROXISOME | 0 | 6.00E-04 | 0.001 |
| KEGG_GLYCOSYLPHOSPHATIDYLINOSITOL_GPI_ANCHOR_BIOSYNTHESIS | 0 | 0.018404 | 0.042 |
| KEGG_BUTANOATE_METABOLISM | 0.006073 | 0.031466 | 0.086 |
| KEGG_AMINOACYL_TRNA_BIOSYNTHESIS | 0.001934 | 0.02441 | 0.089 |
| KEGG_PROPANOATE_METABOLISM | 0.005976 | 0.031557 | 0.131 |
| KEGG_CITRATE_CYCLE_TCA_CYCLE | 0.005906 | 0.041643 | 0.177 |
| KEGG_OXIDATIVE_PHOSPHORYLATION | 0.013807 | 0.04581 | 0.21 |
| KEGG_PYRUVATE_METABOLISM | 0.006135 | 0.040942 | 0.215 |
| KEGG_BASE_EXCISION_REPAIR | 0.007463 | 0.036393 | 0.215 |
| KEGG_TERPENOID_BACKBONE_BIOSYNTHESIS | 0.008081 | 0.03434 | 0.22 |
| KEGG_VALINE_LEUCINE_AND_ISOLEUCINE_DEGRADATION | 0.021739 | 0.036111 | 0.246 |
| KEGG_RIBOSOME | 0.006024 | 0.041045 | 0.29 |
